# FGF Signaling Pathway: A Key Regulator of Stem Cell Pluripotency

**DOI:** 10.3389/fcell.2020.00079

**Published:** 2020-02-18

**Authors:** Majid Mossahebi-Mohammadi, Meiyu Quan, Jin-San Zhang, Xiaokun Li

**Affiliations:** ^1^School of Pharmaceutical Sciences and International Collaborative Center on Growth Factor Research, Wenzhou Medical University, Wenzhou, China; ^2^Institute of Life Sciences, Wenzhou University, Wenzhou, China

**Keywords:** FGF, stem cells, pluripotency, self-renewal, transcription factor

## Abstract

Pluripotent stem cells (PSCs) isolated *in vitro* from embryonic stem cells (ESCs), induced PSC (iPSC) and also post-implantation epiblast-derived stem cells (EpiSCs) are known for their two unique characteristics: the ability to give rise to all somatic lineages and the self-renewal capacity. Numerous intrinsic signaling pathways contribute to the maintenance of the pluripotency state of stem cells by tightly controlling key transcriptional regulators of stemness including sex determining region Y box 2 (Sox-2), octamer-binding transcription factor (Oct)3/4, krueppel-like factor 4 (Klf-4), Nanog, and c-Myc. Signaling by fibroblast growth factor (FGF) is of critical importance in regulating stem cells pluripotency. The FGF family is comprised of 22 ligands that interact with four FGF receptors (FGFRs). FGF/FGFR signaling governs fundamental cellular processes such as cell survival, proliferation, migration, differentiation, embryonic development, organogenesis, tissue repair/regeneration, and metabolism. FGF signaling is mediated by the activation of RAS – mitogen-activated protein kinase (MAPK), phosphatidylinositol-4,5-bisphosphate 3-kinase (PI3K)-AKT, Phospholipase C Gamma (PLCγ), and signal transducers and activators of transcription (STAT), which intersects and synergizes with other signaling pathways such as Wnt, retinoic acid (RA) and transforming growth factor (TGF)-β signaling. In the current review, we summarize the role of FGF signaling in the maintenance of pluripotency state of stem cells through regulation of key transcriptional factors.

## Introduction

Pluripotent stem cells (PSCs) display two remarkable characteristics. Firstly, they are capable of giving rise to every somatic cell originating from all three germ layers (endoderm, mesoderm, and ectoderm) but not the extraembryonic tissues. Secondly, they are characterized by their ability to undergo unlimited self-renewal. PSCs include embryonic stem cells (ESCs) derived from inner cell mass (ICM) of the blastocysts, in both mouse and human (Evans and Kaufman, 1981; Martin, 1981; Thomson et al., 1998), induced pluripotent stem cells (iPSCs) produced by the reprograming of somatic cells from mouse and human by means of the coercive expression of octamer-binding transcription factor 4 (Oct4; Pou5F1), sex determining region Y box 2 (Sox2), krueppel-like factor 4 (Klf4), and c-Myc (Takahashi and Yamanaka, 2006; Takahashi et al., 2007; Yu et al., 2007) and post-implantation epiblast-derived stem cells (EpiSCs). Additionally, PSCs can also be obtained by transferring the nucleus of a somatic cell into an enucleated oocyte, a process called somatic cell nuclear transfer (SCNT) (Gurdon, 1962).

At least two pluripotent states have been described, the naïve or ground state and the primed state (Nichols and Smith, 2009). Mouse ESCs (mESCs) are considered to be at the naïve state while post-implantation mouse EpiESCs (mEpiSCs), human ESCs (hESCs) and human iPSCs (hiPSCs) are in the primed phase (Brons et al., 2007). It has been reported that mEpiSCs display similar properties to hESCs (Davidson et al., 2015). These similarities are defined in many aspects including dependence on activin A and fibroblast growth factor 2 (FGF2) signaling pathway (Li and Belmonte, 2017), their transcriptional and epigenetic profiles, random X-chromosome inactivation, culture requirements and morphology and their differentiation capacities (Brons et al., 2007; Tesar et al., 2007). Recently, numerous attempts have been made to generate human naïve PSCs by the resetting of primed PSCs (Hanna et al., 2010; Gafni et al., 2013; Takashima et al., 2014), as well as somatic cellular reprograming (Liu et al., 2017; Kilens et al., 2018; Giulitti et al., 2019). It has also been reported that hESCs isolated from preimplantation ICM could be cultured *in vitro* to acquire a naïve pluripotent state (Guo et al., 2016). The main concern about naïve hPSCs is the lower passage number compared to primed hESCs, which might be caused by chromosomal instability. Several hESC lines presented with an abnormal karyotype in higher passages, leading to the notion that naïve hPSCs may be more prone to genomic instability in culture (Eguizabal et al., 2019). Because of the chromosomal instability, most of the reports could not maintain the nhPSCs.

**FIGURE 1:**
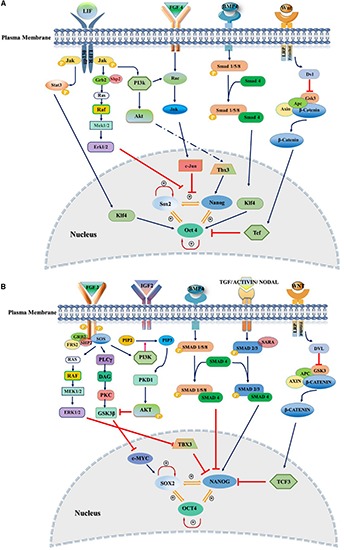
Extrinsic signaling pathways governing stemness of pluripotent stem cells. Pluripotency and self-renewal characteristics of stem cells modulated by positive or negative regulation of SOX2, NANOG, and OCT3/4 by various signaling pathways in the nucleus of both mouse and human. **(A)** Mouse naïve pluripotency mainly controlled by LIF/STAT3, BMP4, Wnt/β-Catenin, and FGF4/ERK signaling pathways. LIF maintains pluripotency through binding to its receptor, gp130/LIFR, followed by activation of JAK/STAT3. Phosphorylated STAT3 interacts with KLF4 and maintains the pluripotency through OCT3/4. BMP4/SMAD signaling controls core transcriptional TFs through interaction with KLF4. FGF4/ERK signaling promotes differentiation of mESCs through JNK/c-JUN and MEK/ERK pathways as downstream regulators. **(B)** Primed state of pluripotency in mEpiSCs, hESC, and hiPSCs is mainly controlled by FGF2/ERK and TGFβ/Activin/Nodal pathways. FGF2 acts through PI3K/AKT, PLCγ and MEK/ERK. TGF/SMAD pathway directly controls pluripotency through interaction with NANOG. IGF2 binding to IGF1R activates PI3K/AKT pathway and regulates stemness by interaction with SOX2. Inhibitors and activators of signaling pathways showed by red blunt-headed and dark blue arrows, respectively.

Self-renewal and pluripotency of stem cells are governed by extrinsic signals mediated by an endogenous pluripotency gene regulatory network consisting of a set of core transcription factors (TFs), such as Oct4, Sox2, and Nanog. TFs interactions contribute to regulate genomic functions by establishing both negative and positive feedback loops and the transcription by binding to specific sites on genomic DNA and recruitment of activators and repressors to modulate the transcriptional machinery (Rizzino, 2009; Young, 2011; Theunissen and Jaenisch, 2014). Maintaining stemness of mouse and human PSCs relies on distinct extrinsic signaling pathways including leukemia inhibitory factor (LIF)/signal transducer and activator of transcription 3 (STAT3), FGF/extracellular signal-regulated kinase (ERK) pathway, phosphoinositide 3-kinase (PI3K)/AKT, Wnt/glycogen synthase kinase 3 (GSK3), and transforming growth factor-beta (TGF-β) signaling (Figure 1). Recently, it has been reported that temporary low dose exposure to retinoic acid (RA) restrains hESC differentiation through blocking the Wnt canonical pathway. This treatment results in retaining stem cell ground state pluripotency (De Angelis et al., 2018). Many reports illustrated that activated FGF signaling plays a pivotal role in sustaining stem cells capabilities through the activation of RAS – mitogen-activated protein kinase (MAPK), PI3K/AKT, phospholipase C gamma (PLCγ) and STAT. In addition, the crosstalk with other pathways such as Wnt, RA, and TGF-β signaling has been reported (Stavridis et al., 2010; Fathi et al., 2017; Tang et al., 2019; Figure 1).

Understanding the mechanisms underlying the pluripotency of PSCs as well as studying how this unique property is retained, are essential not only for the elucidation of mammalian embryogenesis and cellular commitment but also for establishing successful stem-cell-based therapies for regenerative medicine along with disease modeling and drug discovery.

In this review, we discuss the signaling pathways necessary to maintain the pluripotency of the stem cells with a focus on the role of FGF members.

## Maintaining Primed and Ground State Pluripotency Through Extrinsic Signaling Pathways

Pluripotency maintenance in ESCs and iPSCs are provided by inhibiting the signaling pathways governing the differentiation potential of the stem cells (Akberdin et al., 2018).

**FIGURE 2:**
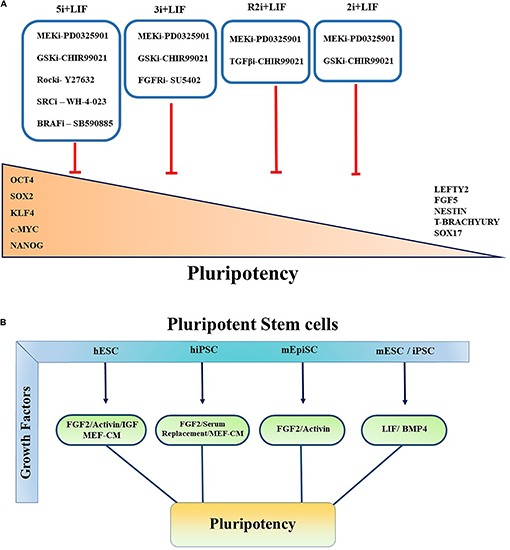
Controlling pluripotency using small molecule inhibitors and growth factors. Primed and Naïve pluripotent stem cells cultured in various conditions in the presence of small molecule inhibitors and multiple growth factors to govern the self-renewal, pluripotency, and conversion of primed to naïve pluripotent stem cells. **(A)** All the inhibitors indicated by “i.” Red blunt-headed arrow demonstrates the inhibition of differentiation and expression of lineage-committed markers such as Nestin, FGF5, Sox17, Lefty, T-Brachyury, and Lefty2. **(B)** Pluripotent characteristics of PSCs controlled by multiple growth factors. Key growth factors contributing to maintainance of pluripotency of PSCs are summarized.

Initially, mouse ESCs (mESCs) were established by co-culturing the cells isolated from the ICM from inbred 129 strain mice with mitotically inactivated mouse embryonic fibroblasts (MEFs) containing fetal calf serum (FCS) (Evans and Kaufman, 1981). Since then, various culture conditions and xeno-feeder-free defined culture medium such as mTeSR, Essential 8 (E8) medium and hESF9 were explored and introduced to sustain the pluripotency state of PSCs (Ludwig et al., 2006; Chen et al., 2011; Figure 1). The culture conditions used combinations of different small molecules and growth factors to control the extrinsic signaling pathways which are known to play critical roles in differentiation of stem cells (Figures 2A,B).

Leukemia inhibitory factor acting through Janus kinase (JAK)/STAT3 signaling pathway has been described as a replacement to the MEFs (Smith et al., 1988; Williams et al., 1988). Recently, it has been demonstrated that LIF removal leads to reversible conversion of mESCs from naïve state to four FGF receptors (FGFR)/ERK-committed early differentiation state. This change is mediated through direct phosphorylation of mammalian target of rapamycin (mTOR) or phosphorylation of a negative regulator of mTOR-trophoblast stem cell (TSC)1/TSC2 proteins. The resulting state has some features characteristic of the primed pluripotency (Cherepkova et al., 2016). Later it has been reported that Bone morphogenetic proteins (BMPs) through the SMAD-inhibitor of differentiation (Id) pathway with LIF could retain the stem cell self-renewal and differentiation potential in mESCs (Morikawa et al., 2016).

Nonetheless, results from various studies revealed considerable differences in controlling self-renewal between human and mouse PSCs. For instance, LIF signaling is dispensable for maintaining pluripotency within hESCs (Zhang et al., 2019). Additionally, TGFβ/activin/nodal signals via SMAD2/3 are also associated with pluripotency and required for the maintenance of the primed hESCs and mouse epiblast (Guzman-Ayala et al., 2004; James et al., 2005).

Furthermore, it has been shown that inhibiting MAPK/ERK signaling pathway activation downstream of FGF signaling using small molecule inhibitors increased ESC stability and stemness. Kunath et al. (2007) demonstrated that FGF4/ERK signaling pathway activation is crucial for mESC multi-lineage differentiation. Various combinations with MEK inhibitor have been reported to maintain the ground state pluripotency of PSCs and to convert the primed human pluripotent cells to naïve PSCs. Blocking GSK3 with its small molecular inhibitor (GSKi; CHIR99021) and MEK inhibitor (MEKi; PD0325901) activity, known as “2i,” together with LIF promotes the establishment and unrestricted expansion of mouse ESCs in the naïve state (Ying et al., 2008). GSK3 acts through Wnt/β-catenin signaling pathway by regulating TCF/LEF TFs (Kelly et al., 2011; Sokol, 2011; Figure 2A). In the canonical Wnt pathway, GSK3 phosphorylates β-catenin and causes its degradation. The inhibition of GSK3 leads to the stabilization of β-catenin and its nuclear translocation (Lee et al., 2019). Shuttling of β-catenin to the nucleus results in self-renewal via revoking the repressor activity of TCF3 (Martello et al., 2012), which has been shown to control ESCs self-renewal by repression of key naïve TFs ESRRB, NANOG, KLF4, and TBX3 (Yi et al., 2008; Martello et al., 2012).

Alternatively, another culture condition has been introduced based on PD0325901 and TGFβ1 receptors inhibitor (TGFi; SB431542) called R2i. This medium formulation results in higher efficacy in the generation of naïve mESCs through the increment of BMP4 signaling pathway even from single blastomeres (Hassani et al., 2014). It has been determined that using 3i chemicals which is comprised of CHIR99021, PD184352 (ERK1/2 inhibitor) and SU5402 (FGF receptor inhibitor) not only maintains the ground state pluripotency but also results in higher differentiation potential and iPSC reprograming efficiency (Ying et al., 2008; Nishihara et al., 2019). Several other culture conditions have also been proposed to sustain the metastability of naïve hESCs or generating naïve pluripotent cells from primed state cells. It has been observed that following resetting of human PSCs by exogenous transgenes KLF2 and Nanog, the naïve state can be effectively sustained in a medium containing inhibitors of GSK3 and MAPK/ERK pathway supplemented with LIF and protein kinase C (PKC) inhibitor (Gö6983). In another study, Theunissen team reported that a combination of five inhibitors targeting MEKi, GSK3i, BRAFi (SB590885), ROCKi (Y27632), and SRCi (WH-4-023) led to the formation of a new generation of naïve human PSCs. They showed that the addition of FGF2 and Activin (5i/L/FA) further enhanced the expression of Oct4, thereby allowing the reversion from primed to naïve state of pluripotency (Theunissen et al., 2014; Figure 2A).

Transcriptional regulators are indispensable factors for pluripotency maintenance for both mESCs and hESCs. Besides the most reported transcriptional regulators such as Nanog, Oct4, and Sox2, FGF shows a spectacular perspective in signal transduction studies and has been reported as a core signal activator of Nanog expression (Vallier et al., 2005; Greber et al., 2007; Singh et al., 2012).

## Fgf Signaling Pathway in PSCS

Fibroblast Growth Factor (FGF) is a family of 22 known polypeptides which are structurally and functionally relevant in biochemical characteristics. They generally share 30–50% amino acid sequence homology. These polypeptides have two conserved cysteine residues and are characterized by a high affinity to heparin/heparin sulfate (HS) (Geary and LaBonne, 2018). Among all the FGFs, their two prototypic FGF members with different isoelectric points are called acidic FGF (aFGF/FGF1) and basic FGF (bFGF/FGF2), respectively. FGF1 has been reported to highly expressed in the brain, retina, bone matrix and osteosarcomas as well as cardiac tissue, while FGF2 is found abundantly in many tissues, including the pituitary gland, neural tissue, adrenal cortex, corpus luteum, and placenta (DePhillips and Lenhoff, 2004).

Fibroblast growth factors are a family of multifunctional proteins, which exert a plethora of effects during embryonic development and homeostasis. All FGFs, except FGF11, FGF12, FGF13, and FGF14 act through their specific receptors (FGFRs), including mitogenesis and non-mitogenic effects (Yun et al., 2010). The FGF ligands elicit their activity by using four transmembrane tyrosine kinase receptors FGFR1, FGFR2, FGFR3, and FGFR4 (Hui et al., 2018). The activated FGFRs will transduce the signals through three dominant pathways including RAS/MAPK, PI3k/AKT, and PLCγ (Yun et al., 2010; Li et al., 2016).

Fibroblast growth factors orchestrate the pluripotency process. They do so by controlling the expression of subsets of genes expressed by pluripotent blastula cells. They regulate essential biological processes such as survival, proliferation, differentiation, and migration during mammalian cell development. FGF signaling is modulated as the stem cells are losing their pluripotency characteristics and begin lineage restriction (Geary and LaBonne, 2018). Engagement of FGF ligands to their specific receptors leads to phosphorylation of various effectors as well as post-translational protein modifications in cells (Bendall et al., 2007; Mason, 2007). Tyrosine phosphorylation of various proteins at specific residues subsequently leads to the activation of PI3K and cRAF and AKT (Zhou et al., 2009). Activation of either the PI3K or AKT pathway elicits a direct yet distinct effect on pluripotency (Singh et al., 2012). AKT arbitrates apoptosis inhibition via mTOR signaling in hESCs, which will enhance proliferation. On the other hand, PI3K activation is necessary to activate the differentiation process in hESCs. Collectively, these observations strongly suggest that FGF signaling acts effectively via PI3K/AKT in hESCs pluripotency.

AKT activation leads to ERK signaling inhibition, which causes differentiation in hESCs (Singh et al., 2012). This inhibition may occur by AKT binding to cRAF and ERK deactivation by blocking its phosphorylation (Stavridis et al., 2007).

Pluripotent stem cells display and require MAPK signaling, whereas PI3K/AKT signals increase as pluripotency is restricted. Such signals are mandatory for the progressive transition of these cells to specific lineage-restricted states.

It is to be noted that even though the cellular origin of both hESC and mESCs are blastocysts, and are both subordinated to the expression of similar transcriptional factors, they nonetheless necessitate diverse cell culture conditions to allow *in vitro* self-renewal and pluripotency.

## Effective Members of the Fgf Family During Pluripotency Maintenance

The FGF signaling pathway plays distinguished roles in numerous cellular functions including embryonic development, tissue regeneration, wound healing, and metabolic homeostasis (Li, 2019). FGFs are potent regulators of differentiation and proliferation of different types of tissue-specific stem cells including hematopoietic stem cells, neural, spermatogonial, prostate, and bone marrow-derived mesenchymal stem cells (Huang et al., 2015; Tian et al., 2019). Many members of the FGF family including FGF2, FGF4, FGF6, FGF7, FGF8, and FGF9 have been reported to impact the stemness of PSCs. Among them, it is clearly described that FGF2 and FGF4 are highly pertinent to maintain mouse and human stem cells in the undifferentiated state.

## Fgf2

It has been previously reported that hESCs expressed all FGFRs. Interestingly, FGF2 is one of the pleiotropic ligands that signal through all FGFRs (Ding et al., 2010). Although hiPSC expressed all four FGF receptors, it has been reported that binding of FGF2 to FGFR1, as the most important receptor, activates downstream signaling including MAPK/ERK, PLCγ, and PI3K/AKT pathways (Nakashima and Omasa, 2016). FGF2 preserves the unique characteristics of stem cells through interaction with PI3K/AKT, PLCγ, ERK1/2, and JAK/STAT pathways by activation of Activin A (Feng, 2007). In another study, It has been shown that FGF2 leads to the secretion of Inhibinβ-B, Gremlin 1 and FGF7 from MEF feeder layer. All of these secreted proteins are involved in sustaining the pluripotency state in hESCs cultured on feeder layer or under feeder-free condition (Diecke et al., 2008). Inhibinβ-B with Activin B together leads to activation of smad 2/3 which is essential for retaining stemness of the hESCs (Besser, 2004). In addition, Gremlin 1 acts as an inhibitor of BMP signaling to maintain the undifferentiated state of pluripotency (Xu et al., 2005).

Moreover, the interaction between hESC and autologously derived human ES cell fibroblast-like cells in conditioned medium governs self-renewal of hESC by secreting TGFβ and insulin-like growth factor II (IGF-II) upon FGF2 stimulation (Bendall et al., 2007). Interestingly, FGF2 at high concentration (100 ng/ml) supports hESC self-renewal in the absence of MEF or conditioned medium through inhibition of BMP signaling (Levenstein et al., 2006). PI3K/AKT acts downstream of the FGF signaling and is controlled by Protein arginine methyltransferase 8 (PRMT8) through interaction with the regulatory subunit of PI3K (P85). This interaction results in the enhancement of AKT activity instead of the MEK/ERK pathway. PRMT8/PI3K/AKT axis maintains pluripotency of hESC as well as mesodermal differentiation via the regulation of Sox2 (Jeong et al., 2017). In a recent study, Haghighi et al. (2018) showed that among the downstream mediators of FGF2 signaling pathways, the MAPK pathway plays a pivotal role in maintaining the pluripotency of hiPSCs. They demonstrated that following withdrawal of FGF2, the activity of NRAS-RAF-MEK-ERK declined, while the AKT signaling pathway as one of the downstream of the FGF2 remained unchanged (Haghighi et al., 2018). In another study, it has been shown that the C5a complement member supported the pluripotency of hESC after removal of FGF2 through the ERK1/2 signaling pathway (Hawksworth et al., 2014). Given all these reports, it is clear that FGF2 is mandatory for restraining the pluripotency state of hiPSCs and hESCs (Figure 1B).

## Fgf4

Expression of FGF4 is restricted to undifferentiated embryonic stem (ES) cells and embryonal carcinoma (EC) cell lines. FGF4 expression has not been reported in differentiated cells (Hebert et al., 1991; Basilico and Moscatelli, 1992; Rappolee et al., 1994). In stem cells, a distally localized enhancer controls *FGF4* gene expression. This enhancer contains consensus octamer-binding sites and controls positive regulation in EC and ES cells. The Sox2/Oct-3/4 complex is tonic for normal pluripotent cell development and maintenance. The complex can bind to the *FGF4* enhancer and promote transcriptional activation of *FGF4* (Rappolee et al., 1994; Yuan et al., 1995). Oct-3/4 mRNA is expressed in both human and mouse oocytes and blastocysts. The combination of Oct-3/4 and FGF4 expression is essential for mouse embryo development in the preimplantation stage (Niswander and Martin, 1992; Niwa et al., 2000). However, in post-implantation mouse embryos, FGF4 and Oct-3/4 are expressed in distinct regions as well as in overlapping regions. FGF4 expression is essential for maintenance of pluripotency, but its expression depends on the presence of Oct-3/4 as the regulator. Also, in the presence of RA, FGF4 promotes the differentiation of ES cells into primitive ectoderm. FGF4 addition to the culture medium increases the number of differentiated cells, mostly with many of the properties of parietal extraembryonic endoderm. The inactivation of the *FGF4* gene, particularly at the initial stage of ES cell differentiation, leads to impaired lineage formation. In mouse undifferentiated ES cells, FGF4 causes the activation of ERK1/2 signaling cascade. FGF4 inhibition restricts the differentiation of ES cells (Kunath et al., 2007).

Fibroblast growth factor 4 signaling disruption antagonizes neural and mesodermal induction in ES cells. Moreover, upon FGF4 inhibition, the expression of pluripotency markers Oct-3/4, Nanog, and Rex1 is disrupted. These findings indicate that FGF4-ERK1/2 signaling plays a vital role in neural and mesodermal commitment in ES cells (Nichols et al., 1998; Mayshar et al., 2008; Figure 1A).

The blockade of FGF4-ERK signaling pathway leads to the blockade of trophectoderm differentiation from stem cells. In the mammalian embryo, the activity of Oct-3/4 is vital for the identity of the PSC population. FGF4 presence in the mouse blastocyst culture medium allowed the isolation of TSC population. The combination of FGF4 and TGFβ sustains the continuous proliferation of TSCs. In epiblasts, the TGFβ-related protein Nodal induces *FGF4* expression (Guzman-Ayala et al., 2004). In summary, FGF4 appears to have a unique and vital biochemical characteristic in stem cell proliferation and differentiation through SHP2/SRC/RAS/ERK pathway and maintains the pluripotency of ES cells (Yang et al., 2006).

## Other Fgfs

Fibroblast growth factor 5 is well known as a mEpiSCs or epiblast marker, its expression is generally associated with differentiation and loss of stemness, therefore FGF5 is hardly detectable in mESCs. mEpiSCs as primed state PSCs, are characterized by expressing markers such as Fgf5, T-Brachyury, Cer1, Otx2, Socs3, Acvr2b, and Lefty whereas mESC expressed Rex1, Klf4, and Nanog (Nichols and Smith, 2011). FGF5 is found in embryoid bodies (EBs) from the 1st day of formation (Shirouzu et al., 2016). During murine development, FGF5 is transiently expressed at different stages (Haub and Goldfarb, 1991). Naïve mESCs have the ability to differentiate into mEpiSCs in the presence of small molecules inhibitors or supplementation with FGF2 and Activin A (Guo et al., 2009; Xue et al., 2011). However, conversion in a reverse way is prohibited due to the epigenetic barriers. It has been reported that FGF5 is expressed during cellular commitment to primitive ectoderm but is not expressed in the ICM (Hayashi et al., 2007) and that autocrine FGF5 may function during gastrulation via preserving the mobility of cells, thereby promoting all three germ layers (Hebert et al., 1991).

It has been described that FGF7 (KGF), in addition to Nicotinamide and Activin A in feeder-free condition, affects the proliferative rate of hESC. It is believed that FGF7 is required for cell proliferation (Beattie et al., 2005). Moreover, FGF7 plays a role in differentiation of PSCs toward thymic epithelial cells (Inami et al., 2011). One of the other FGFs that plays role in pluripotency and during ESCs is FGF8. FGF8 and FGFR1 are expressed in the blastocyst and mutation of either result in post-implantation lethality with impaired axis formation and mesoderm specification (Lanner and Rossant, 2010). In addition, it has been reported that FGF2, FGF4, FGF6, and FGF9 could induce high level of NANOG expression in hESCs (Chen et al., 2012).

## Conclusion

Maintaining self-renewal and differentiation capabilities of PSCs in both naïve and primed stem cells is of prime importance for the future use of these cells in both basic and translational research. Recent advances revealed the significant role of various extrinsic signals in retaining pluripotency of stem cells in both mouse and human. Controlling these signaling pathways with several small molecule inhibitors allows the development of new approaches in conserving pluripotency as well as in the conversion of stem cells from the primed to naïve state. Many studies reported substantial differences between negative and positive extrinsic regulators of pluripotency in mouse and human. Major regulators at the base of these differences are FGF members. In addition to the very well reported role of the FGF signaling pathway in cellular homeostasis, FGF signaling is also required for the maintenance of self-renewal and pluripotency of stem cells in the primed state whereas in naïve stem cells FGFs control the differentiation of these cells toward primitive endoderm. So, with this dual role in the maintenance of pluripotency and differentiation, elucidating the diverse aspects of governing FGF signaling is of critical importance in PSCs especially in iPSCs.

## Author Contributions

MM-M, J-SZ, and XL conceived the study. MM-M, MQ, and J-SZ wrote the manuscript. J-SZ and XL supervised the study and acquired funding.

## Conflict of Interest

The authors declare that the research was conducted in the absence of any commercial or financial relationships that could be construed as a potential conflict of interest.

## Abbreviations

aFGF: acidic fibroblast growth factor
bFGF: basic fibroblast growth factor
EBs: embryoid bodies
EC: embryonal carcinoma
EpiSCs: post-implantation epiblast-derived stem cells
ERK: extracellular signal-regulated kinase
ESC: embryonic stem cell
FGF: fibroblast growth factor
GSK3: glycogen synthase kinase 3
hESC: human embryonic stem cell
hiPSC: human induced pluripotent stem cell
ICM: inner cell mass
iPSC: induced pluripotent stem cell
JAK/STAT: Janus kinase/signal transducers and activators of transcription
KLF4: krueppel-like factor 4
LIF: leukemia inhibitory factor
MAPK: mitogen-activated protein kinase
MEF: mouse embryonic fibroblast
mESC: mouse embryonic stem cell
mTOR: mammalian target of rapamycin
OCT4: octamer-binding transcription factor 4
PI3K: phosphatidylinositol-4,5-bisphosphate 3-kinase
PLC γ: phospholipase C gamma
PSC: pluripotent stem cell
RA: retinoic acid
SOX2: sex determining region Y box 2
TF: transcription factor
TGF: transforming growth factor
TSCs: trophoblast stem cells.
